# Weekday of Surgery Affects Postoperative Complications and Long-Term Survival of Chinese Gastric Cancer Patients after Curative Gastrectomy

**DOI:** 10.1155/2017/5090534

**Published:** 2017-04-18

**Authors:** Rong Li, Ai-min Leng, Ting Liu, Yan-wu Zhou, Jun-xian Zeng, Xiao-ming Liu, Ting-zi Hu, Xiao-xia Jiang, Lin-fang Zhang, Can-xia Xu

**Affiliations:** ^1^Department of Gastroenterology, Third Xiangya Hospital of Central South University, Changsha, Hunan 410013, China; ^2^Hunan Key Laboratory of Nonresolving Inflammation and Cancer, Changsha, Hunan 410013, China; ^3^Department of Gastroenterology, Xiangya Hospital of Central South University, Changsha, Hunan 410008, China; ^4^Department of Thoracic Surgery, Xiangya Hospital of Central South University, Changsha, Hunan 410008, China; ^5^Department of General Surgery, Third Xiangya Hospital of Central South University, Changsha, Hunan 410013, China

## Abstract

Many factors have been reported to affect the long-term survival of gastric carcinoma patients after gastrectomy; the present study took the first attempt to find out the potential role of weekday carried out surgery in the postoperative prognosis of gastric cancer patients. 463 gastric cancer patients have been followed up successfully. Pearson *χ*^2^ test was used for univariate analyses. Survival curves were constructed by using Kaplan-Meier method and evaluated by using the log-rank test. The Cox proportional hazard regression model was used to find out the risk factors, and subgroup analysis was conducted to rule out confounding factors. We found that the patients who underwent gastrectomy on the later weekday (Wednesday–Friday) more easily suffered from a higher postoperative morbidity. Weekday of surgery was one of the independent indicators for the prognosis of patients after gastric cancer surgery. However, the role of weekday of surgery was significantly weakened in the complications group. In conclusion, surgery performed in the later weekday was more likely to lead to increased postoperative complications and an unfavorable role in prognosis of Chinese gastric cancer patients after curative gastrectomy.

## 1. Introduction

With a mortality of 498.0/100,000, gastric cancer has been regarded as the forth leading cancer-related lethal disease and ranks as one of the 5 most commonly diagnosed cancers in China [1]. Though early diagnosis of gastric carcinoma has gained great progress because of the rapid advancement in the endoscopic technique, the outcome of stomach malignancy continues to be unsatisfactory [1]. Consequently, investigators are engaged in finding out which controllable factors really affect the prognosis, thus to improve the current dismal situation of gastric cancer. Long-term outcome after gastrectomy relies on several universal well-known factors such as tumor differentiation, numbers of involved lymph nodes, TNM stage, operation technique, and adjuvant therapy [2, 3]. Even the extreme body mass index and hypoproteinemia are reported to indicate a poorer prognosis [4–6]. Currently, gastrectomy is still the basic and widely accepted treatment for curable gastric carcinoma. Though surgery always produces more satisfactory outcomes in experienced hands and specialized centers [7], its accompanying postoperative complications are reported to increase the postoperative quality and survival differently [8–10]. It indicates that control of postoperative complications would significantly improve the disappointing survival.

Interestingly, previous researches have also reported that the weekday choice of surgery did play a significant role in the prognosis after tumorectomy. In an analysis of 4, 133, 346 inpatient admissions for elective operating room procedures, Aylin et al. reported that surgery in the later week or weekend increased postoperative 30-day mortality [11]. The 5-year mortality of curable esophageal cancer was increased after surgery later in the week [12, 13]. However, whether weekday of surgery plays a role in postoperative complications or prognosis of gastric cancer is not clear.

Therefore, the present study was aimed at identifying the potential role of weekday of surgery in postoperative complications as well as the long-term survival after gastrectomy. In this study, we found that patients who underwent surgery on the later weekday (Wednesday to Friday) more easily suffered from an increased postoperative morbidity and a poorer prognosis in gastric cancer than those in the earlier weekday. Taken together, our results indicated that more strict postoperative complications control and proper scheduling would improve the prognosis of gastric cancer.

## 2. Materials and Methods

### 2.1. Patient Selection Criterion

Firstly, we have gained the approval of the Institutional Review Board of Third Xiangya Hospital of Central South University, Hunan, China. From January 1, 2012, we retrospectively collected the medical and clinicopathological information of the patients who were diagnosed with gastric carcinoma and underwent gastrectomy during the period from January 1, 2004, to January 1, 2010, at the Department of Gastrointestinal Surgery, Xiangya Third Hospital. The details for recruited patients have referred to the previously described methods [14]. The selection criteria were as follows: (1) patient was diagnosed with gastric carcinoma histologically confirmed by at least 2 pathologists, without distant metastasis and received curative gastrectomy; (2) lymphadenectomy was performed with R0 margin if necessary; (3) the details regarding clinicopathological features, postoperative complications, and follow-up information were available and intact; (4) life span after operation was more than 60 days. As a consequence, there were 463 patients enrolled in our analysis. The clinicopathological data were collected in detail from the original medical records as summarized in the Table 1; they were grouped by clinical, pathological, and therapy-related characters. Additionally, the tumor-node-metastases (TNM) stage was classified according to American Joint Committee on Cancer, AJCC 7th edition [15]. All the personal information was concealed to ensure the privacy. The design and methods of this study were in accordance with the requirements of related regulations and procedures (such as GCP, ICH-GCP) as well as the ethical principles. The IRB of Third Xiangya Hospital, Central South University, has approved the research to be conducted and the approval number was 2016-S232. All authors had access to information that could identify individual participants during or after data collection.

### 2.2. Complications

Any deviation from the normal course within 30 days after operation was seen as a postoperative complication, which was recorded in detail (medical, surgical, infectious complications, etc.). According to the revised Clavien-Dindo classification system [16], the concreteness was as follows: Grade I complications include any deviation from the uneventful postoperative course without the need for pharmacological treatment or surgical, endoscopic, and radiological interventions, except the drugs as follows: antipyretics, antiemetics, analgesics, electrolytes, diuretics, and physiotherapy. Wound infections and nutritional support were also included. Grade II complications could be treated solely by drugs, blood transfusion, and parenteral nutrition. Grade III complication required endoscopic, radiological, or surgical intervention. Grade IV included life-threatening complications (including CNS complications) which required intensive-care unit management. Grade III or Grade IV altogether was regarded as severe complications. Grade V meant death of the patient, which was excluded from our analysis. The details were displayed in Table 2.

### 2.3. Follow-Up

The objects enrolled were followed up every 3 months during the first postoperative year and at least 6 months afterward for survival and recurrence inquiry. The data were obtained by telephone, letter, message, and return visitation. The follow-up data were calculated from the date of surgery to the death or the end of data collection. The follow-up was stopped on January 1, 2015.

### 2.4. Statistics

The SPSS software (version 16.0, Chicago, IL) and GraphPad prism 5.0 were used to complete all statistical analyses and graphics. To clarify the potential variables affecting the postoperative morbidity, the clinical, pathological, and therapy-related characters were grouped and compared between those who underwent complications or not. A Pearson *χ*^2^ test or Fisher's exact test was used for univariate analyses where appropriate. Survival curves were constructed by using the Kaplan-Meier method and evaluated by using the log-rank test. Subgroup analysis was conducted to rule out confounding factors. The Cox proportional hazard regression model was used to identify factors that were independently associated with overall survival and disease-free survival. The variables with a* p* value less than 0.05 in the univariate analysis were considered potential candidates for the main effects and were evaluated in a stepwise multivariate Cox analysis. In multivariate analysis,* p* values less than 0.05 were considered statistically significant.

## 3. Results

*(1) Patients Who Received Gastrectomy in the Later Weekday Were Prone to Suffering from the Postoperative Complications*. From January, 2004, to January, 2010, a total of 495 consecutive gastric carcinoma patients who underwent gastrectomy were enrolled; among them 32 patients died in 30 days (Grade V, <30 days) after operation, and they have been excluded in this analysis. As shown in Table 1, there were 198 patients took surgery on the earlier weekday (Monday or Tuesday) and 265 patients on the later weekday (Wednesday–Friday). The patients who underwent gastrectomy on the later weekday more easily suffered from a longer operative duration or more operative related blood loss. Moreover, the patients who took gastrectomy on the later weekday had a higher risk of suffering from postoperative complications. However, we found that there was no major difference in distribution of general state (age, gender, smoking history, and BMI), disease-state (comorbidities and ASA grade), tumor state (differentiation, size, serum CEA level, and TNM stage), and therapeutic schedule (operation procedure and adjuvant therapy) between the two groups.

There were about 21.8% (101/463) patients who experienced postoperative complications eventually. 34 patients in the earlier weekday had postoperative complications and 67 in the later weekday. They were classified into 4 groups according to the revised Clavien-Dindo classification system [16] (Grade I, *n* = 45; Grade II, *n* = 28; Grade III, *n* = 17; Grade IV, *n* = 11). Furthermore, we found that the postoperative morbidity was increased gradually from Monday to Friday. Though not statistically significant, the degree of complications severity increased from Wednesday through Thursday to Friday. Moreover, the severe complications (Grade III + Grade IV) mainly focused on Thursday or Friday (Table 2).

*(2) Weekday of Surgery Was Associated with the Long-Term Survival in Gastric Cancer Patients after Gastrectomy*. To assess the potential influential factors in gastric cancer prognosis, Cox proportional hazards regression model was introduced. Univariate analyses (Table 3) displayed that the postoperative 5-year survival was associated with comorbidities, tumor size, operative time, intraoperative blood loss, postoperative serum CRP, TNM stage, adjuvant therapy, and postoperative complications. It is worth mentioning that surgery in the later weekday also promoted an unfavorable prognosis after gastrectomy. Then, the variables with a* p* value less than 0.05 in the univariate analysis were further pooled in a multivariate analysis. On multivariate survival analysis, weekday of surgery (*p* < 0.001), postoperative complications (*p* < 0.001), postoperative serum CRP (*p* = 0.026), and TNM stage (*p* = 0.033) reached significance for overall survival time (OS) (Table 4). Surgery performed in the later weekday decreased the 5-year overall survival rate 1.721 times when compared to the surgery in the earlier weekday. For analysis of disease-free survival time (DFS), weekday of surgery (*p* < 0.001), postoperative complications (*p* < 0.001), postoperative serum CRP (*p* = 0.004), and TNM stage (*p* = 0.027) also reached significance in the multivariate survival analysis Cox proportional hazards regression model. Similarly, the surgery performed in the later weekday increased 5-year relapse rate by 1.693 times (Table 4). It indicated that surgery in the later weekday promoted a worse outcome in gastric cancer patients after gastrectomy.

**Figure 1:**
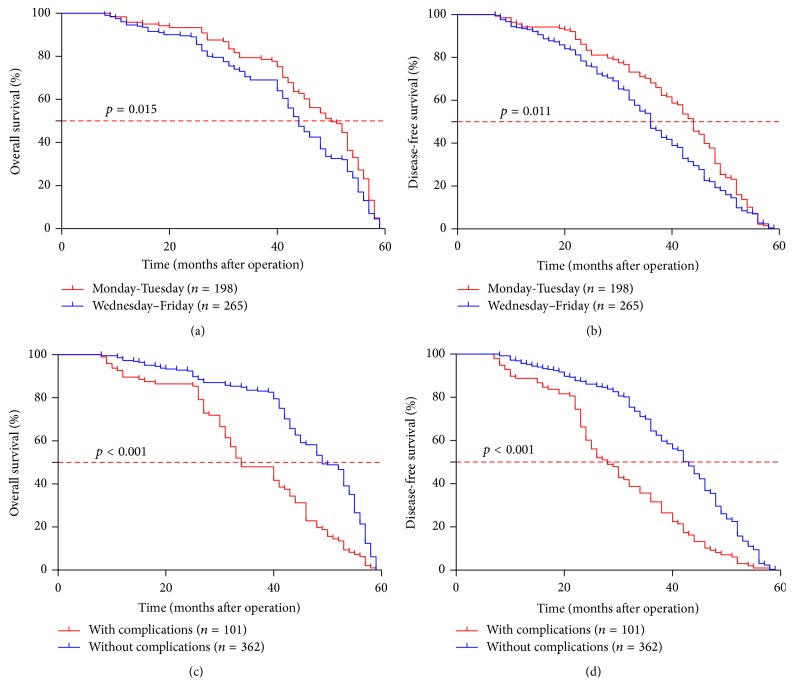
Weekday of surgery or postoperative complications influenced the overall survival time and disease-free survival time after gastrectomy. The 5-year overall survival rate and disease-free survival rate was much lower in the patients who underwent surgery on Wednesday to Friday than those on Monday to Tuesday (a, b). Similarly, the patients with postoperative complications shared a much shorter overall survival and disease-free survival time than those without complications (c, d).

*(3) Patients Who Received Gastrectomy in the Later Weekday or Suffered from Postoperative Complications Had a Shorter OS and DFS*. Moreover, the Kaplan-Meier analysis showed that patients who undertook gastrectomy in the later weekday had a shorter survival time compared to those in Monday or Tuesday (OS: 44 versus 50 months, *p* = 0.015; DFS: 36 versus 44 months, *p* = 0.011; Figures 1(a) and 1(b)). Moreover, compared with the surgery carried out on Monday, the surgery on Friday increased relapse and death rate (HR (hazard ratio) = 1.415, 95% CI, 1.054–1.901 and HR = 1.559, 95% CI, 1.142–2.128), respectively (Table 5). As we all know, it needs more time and energy to complete a surgery for a tumor in advanced TNM stage; we also demonstrated that risk estimates of gastric cancer surgery in the later weekday were evident for tumor in stage I or II, but not for stage III (Supplementary Figure 1 in Supplementary Material available online at https://doi.org/10.1155/2017/5090534).

As shown in the multivariate analysis, the postoperative complication was also an independent indicator for the gastric cancer outcome. Furthermore, gastric cancer patients with postoperative complications had much shorter OS than those without complications (median survival time, 34 versus 49 months, *p* < 0.001; Figure 1(c)) and displayed an increased mortality (adjusted HR = 3.169, 95% CI, 2.388–4.204, Table 4). Similarly, gastric cancer patients with postoperative complications had a shorter DFS (median survival time, 28 versus 43 months, *p* < 0.001; Figure 1(d)) than those without complications and showed an increased postoperative relapse rate (adjusted HR = 2.826, 95% CI, 2.134–3.740, Table 4). Moreover, when postoperative complication was categorized into the 5 degrees, the HR for postoperative relapse rate or mortality gradually increased from Grade 0 to Grade IV (Supplementary Table 1).

Similarly, in order to clarify the potential role of postoperative complication in different TNM stage, we conducted a subgroup analysis, and we found a significant role of postoperative complications in predicting prognosis in stages I and II, but not in stage III (Supplementary Figure 2).

**Figure 2:**
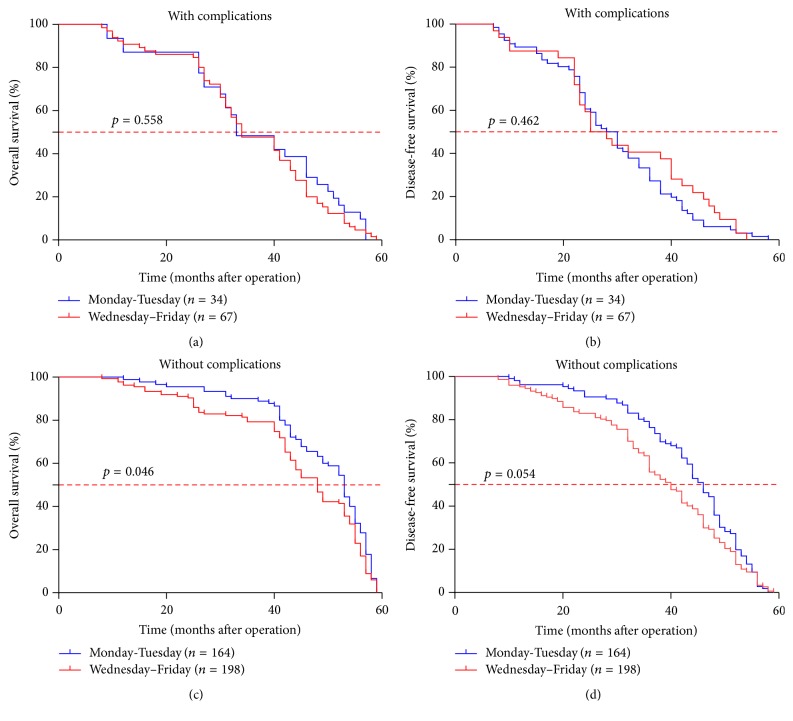
In the patients with postoperative complications, there was no significant difference between surgery on the earlier weekday and the later weekday in overall survival time and disease-free time (a, b). However, the weekday of surgery is still likely to influence the 5-year overall survival rate (c) in the patients without complications but not relapse rate (d).

**Figure 3:**
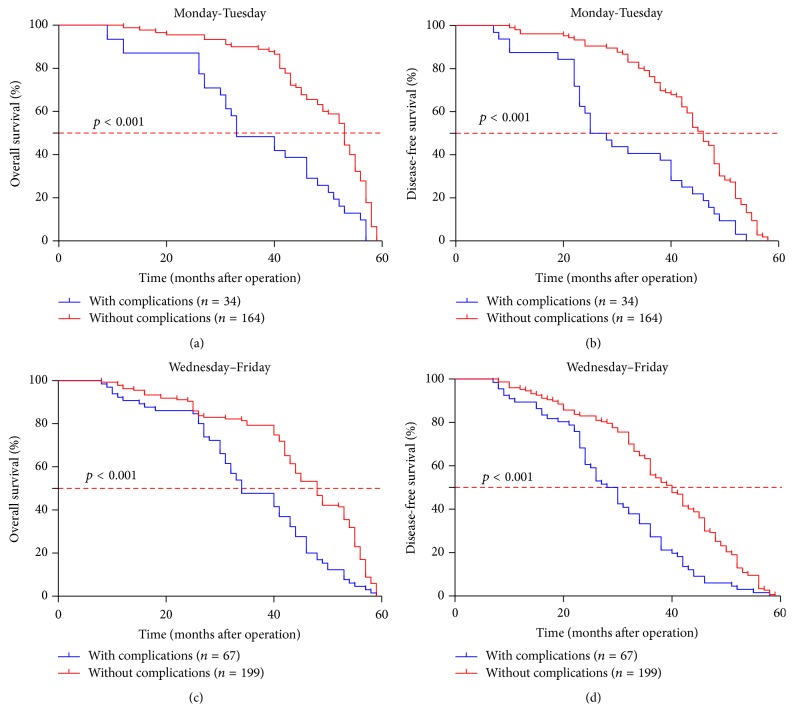
Regardless of weekday of surgery, the postoperative complications have a significant influence on the overall survival time and disease-free time after gastrectomy.

*(4) Surgery Performed in the Later Weekday Might Deteriorate the Long-Term Survival by Promoting the Postoperative Complications*. As noted above, patients who got gastrectomy in the later weekday showed higher risk of suffering from postoperative complication and a poor prognosis (Table 1, Figure 1). To clarify the inner relationship between weekday of surgery and postoperative complications in prognosis, a subgroup analysis was conducted. When complication condition was confined, the effect of weekday of surgery was limited in patients with complication (Figure 2, Supplementary Table 2). However, when we confined the weekday of surgery, we found that the occurrence of postoperative complications promoted a much shorter survival time regardless of the weekday of surgery (Figure 3, Supplementary Table 3). It indicated that the postoperative complication was a valid potential prognostic indicator; it might conceal the effect of the weekday of surgery on prognosis after surgery for gastric cancer to some extent.

## 4. Discussion

Due to the current unsatisfactory outcome of gastric cancer patients, clinical doctors and researchers have put a lot of efforts to improve survival in resectable gastric cancer [17, 18]. Postoperative complications have been reported to not only prolong the postoperation hospitalization duration and increased costs [19], but also promote unfavorable prognosis [8–10, 20]. Surgical complication is tightly related to the quality of the operative technique and the surgeon's case-load; the morbidity is higher in inexperienced hands [7, 21]. As multidisciplinary collaboration could be performed in a general hospital, the occurrence of surgical complications was relatively low in our institution, compared to morbidity from 15.5% to 24.7% [6]. However, in the present study, postoperative complication was also demonstrated to be an independent indicator of poor prognosis after gastric cancer surgery. As discussed previously, postoperative complications commonly promoted a persistent period of immunosuppression and allowed residual tumor cells to survive in the host, which lead to an earlier cancer recurrence [22, 23]. Moreover, excessively activated inflammatory response was also an important aspect. Like in our results, postoperative complications always accompanied with an increased serum CRP, the infectious complications potentiated proinflammatory cytokine cascades, including interleukins 1, 8 and tumor necrosis factor-alpha. These activated cytokines could disable the function and regulation of cytotoxic T-lymphocytes, natural killer cells, and antigen-presenting cells [24–26].

Therefore, identifying factors related to complications would also aid in the successful treatment of gastrectomy patients. Interestingly, we found that the weekday of surgery was associated with the occurrence of postoperative complications. Previously, the “weekend effect” has been proposed that postoperative complications might be less well handled during weekends [27]. Some research has also revealed that surgery in earlier weekday was regarded as beneficial in short term (30 days) after elective surgery [11, 13]. Esophageal cancer surgery was suggested to be carried out earlier in the week, for the increased 5-year mortality of potentially curable esophageal cancer after surgery later in the week [12]. Moreover, our present study has demonstrated that surgery performed in the later weekday was an indicator of the adverse outcome after gastrectomy in gastric cancer patients. To the best of our knowledge, this is the first report to address the potential influence of weekday of gastrectomy in relation to postoperative complications and long-term survival in gastric cancer. Moreover, surgery performed in the later weekday deteriorated the long-term survival by promoting the postoperative complications.

Factors like the age or tumor stage did not influence the choice of operation day in this study; however, the occurrence of postoperative complications and complications severity were affected by the weekday of surgery. All these might be interpreted by the hypothesis that the health care services were of lower quality during the later weekday. Firstly, the surgical precision might be deteriorated to some extent due to the workload of surgeons and surgical team; secondly, the adverse outcome of gastric surgery might be partly influenced by the alertness of the surgeon; thirdly, it was not difficult to deduce that a surgeon would find it much easier to concentrate on exhausting and demanding surgery in the earlier weekday. Above all, this hypothesis gained support from the findings that operative time and operative blood loss were increased in the later weekday slightly but significantly. Inadequate staffing on the following weekend after surgery is indeed one of the factors that should be considered, as complications were more likely picked up later with delayed medical care [27]. Moreover, the degree of complication severity was increased from Wednesday to Friday, compared to Monday. Even so, the effect of weekday of surgery or postoperative complications was weak in the patients with advanced tumor stage for the little chance of cure.

Moreover, from the present analysis, we found that preoperative comorbidity was a significant impact factor for morbidity, as described previously [28]. Beyond that, we also found that various influential factors are reported to increase the occurrence of complications after surgical resection, including old age, abnormal BMI, large tumor size, low serum albumin, longer duration of operation, and advanced tumor stage which was consistent with what has been reported before [9, 29]. Accordingly, our results also showed that smoking had an obvious influence on the occurrence of complications; we could infer that except the sensitive respiratory system, nicotine addiction can easily induce an inflammatory state, thus impairing the body repairing and healing ability [30, 31]. In contrast to the proposed “obesity paradox” [6] that overweight and obesity promoted better outcomes in patients after gastrectomy, our study revealed that patients with high BMI had an higher morbidity after the gastric surgery; the increased rate was mainly due to the fact that excess fat may increase operation difficulty, operative time, and wound fat liquefaction as well, whose effects were significant in our study. Moreover, advanced tumor stage accompanied with more intraoperative blood loss, complicated operative procedure, and intensive shock was demonstrated to facilitate postoperative complications.

In the present study, preoperative comorbidity was responsible for the medical complications, especially in those with diabetes mellitus, which increased the incidence of infectious complications by more than 5 times. Preoperative coexisted active hepatic or nephrotic disease was obligated for most Grade IV complications in our study. Moreover, our results revealed that it was the severity grade of postoperative complications that promoted distinct clinical outcomes, but not complications type. Furthermore, the old age also represented a synergetic role with the postoperative complications in deteriorating the outcome. These results offered crucial clinical implications for selecting appropriate patients for surgery to reduce the postoperative morbidity. In conclusion, our present study indicated that the surgery performed on the later weekday associated with an increased postoperative morbidity and predicted a poor prognosis after gastric cancer surgery. Gastric cancer surgery for more readily surgically curable tumor stages (I-II) was followed by a less postoperative morbidity, relapse, and a better prognosis if conducted in the earlier weekday and the patient was well prepared. Thus, scheduling of gastric cancer surgery properly and a strict perioperative management to decrease the occurrence of postoperative complications might help to improve the prognosis in patients operated on for gastric cancer.

## Supplementary Material

Degree of postoperative complication influenced the overall survival and disease-free survival. The occurance of postoperative complications promoted a much shorter survival time regardless of the weekday of surgery, however, the effect of weekday of surgery was limited in the group with complications. Even so, effect of weekday of surgery or postoperative complications was weak in the patients with advanced tumor stage for the little chance of cure.

## Figures and Tables

**Table 1:** The clinicopathological characters of 463 patients subjected to curative gastrectomy.

Characteristics	*n*	Monday-Tuesday	Wednesday–Friday	*p* value
Gender				
Male	224	102	122	0.26
Female	239	96	143	
Age (year)				
≤65	265	123	142	0.066
>65	198	75	123	
Smoking history				
No	196	80	116	0.506
Yes	267	118	149	
BMI				
Low BMI (<18 kg/m^2^)	175	73	102	0.936
Normal BMI (18–25 kg/m^2^)	247	107	140	
High BMI (>25 kg/m^2^)	41	18	23	
Comorbidities				
No	340	139	191	0.670
Yes	123	49	74	
ASA (0.000)				
I	340	139	191	0.850
II	101	41	60	
III	22	8	14	
Differentiation				
Undifferentiated	23	10	13	0.611
Differentiated	440	188	252	
Tumor size				
≤5 cm	322	136	186	0.76
>5 cm	141	62	79	
Hypoproteinemia				
No	250	115	135	0.133
Yes	213	83	130	
Increased CEA				
No	263	108	155	0.448
Yes	200	90	110	
T classification				
T1	13	5	8	0.345
T2	135	57	78	
T3	276	114	162	
T4	39	22	17	
N classification				
N0	198	96	102	0.158
N1	195	77	118	
N2	53	18	35	
N3	17	7	10	
TNM stage				
I	73	34	39	0.561
II	321	132	189	
III	69	32	37	
Procedure (whether laparoscopic-assisted)				
No	397	167	230	0.502
Yes	66	31	35	
Medical team				
Group 1	167	69	98	0.757
Group 2	155	70	85	
Group 3	141	59	82	
Chemotherapy				
No	106	49	57	0.412
Yes	357	149	208	
Operation time				
≤240 min	354	164	190	**0.006**
>240 min	109	34	75	
Intraoperative blood loss				
≤500 ml	391	176	215	**0.027**
>500 ml	72	22	50	
Postoperative complications				
Without	362	164	198	**0.041**
With	101	34	67	
Postoperative complications				
Grade 0	362	164	198	0.072
Grade I	45	19	26	
Grade II	28	9	19	
Grade III	17	5	12	
Grade IV	11	1	10	
Postoperative CRP				
Normal CRP	285	121	164	0.923
Increased CRP	178	77	101	

**Table 2:** Patients who took surgery on the later weekday were more inclined to get severe complications (Grade III + Grade IV).

	Grade 0	Grade I	Grade II	Grade III	Grade IV
Monday	107	11	4	5	0
Tuesday	57	8	5	0	1
Wednesday	60	4	4	5	0
Thursday	60	7	5	3	6
Friday	78	15	10	4	4

**Table 3:** Univariate cox analysis of overall and disease-free survival in 463 patients after gastrectomy.

Characteristics	Overall survival		Disease-free survival	
	HR (95% CI)	*p* Value	HR (95% CI)	*p* Value
Gender (female/male)	1.190 (0.956–1.483)	0.120	1.158 (0.939–1.428)	0.171
Age (year) (>65/≤65)	1.126 (0.903–1.405)	0.293	1.153 (0.933–1.424)	0.187
BMI (abnormal BMI/normal BMI)	1.093 (0.878–1.361)	0.426	1.079 (0.875–1.331)	0.477
Smoking history (yes/no)	1.008 (0.808–1.258)	0.945	1.015 (0.821–1.254)	0.893
Comorbidities (with/without)	1.196 (0.936–1.527)	0.152	1.282 (1.018–1.616)	**0.035**
ASA (III/I + II)	1.277 (0.784–2.082)	0.326	1.564 (0.996–2.457)	0.052
Tumor Size (≥5 cm/<5 cm)	1.180 (0.932–1.494)	0.169	1.320 (1.057–1.649)	**0.015**
Tumor differentiation (yes/no)	0.818 (0.502–1.333)	0.420	0.856 (0.526–1.394)	0.533
Increased serum CEA (yes/no)	0.914 (0.732–1.140)	0.425	0.960 (0.777–1.186)	0.704
Hypoalbuminemia (yes/no)	1.032 (0.828–1.286)	0.779	0.971 (0.787–1.198)	0.783
Laparoscopic-assisted (yes/no)	0.883 (0.640–1.218)	0.448	0.952 (0.705–1.284)	0.745
Operation time (≥240 min/<240 min)	1.653 (1.295–2.112)	**<0.001**	1.495 (1.178–1.896)	**0.001**
Operative blood loss (≥500 ml/<500 ml)	1.604 (1.212–2.125)	**0.001**	1.509 (1.149–1.983)	**0.003**
T classification		** <0.001**		**<0.001**
(T2/T1)	1.723 (0.697–4.260)	0.239	1.239 (0.574–2.678)	0.585
(T3/T1)	2.869 (1.181–6.967)	**0.020**	2.042 (0.962–4.334)	0.063
(T4/T1)	4.834 (1.889–12.366)	**0.001**	3.105 (1.378–6.993)	**0.006**
N classification		**<0.001**		**<0.001**
(N1/N0)	2.005 (1.571–2.558)	**<0.001**	2.280 (1.801–2.886)	**<0.001**
(N2/N0)	2.184 (1.521–3.136)	**<0.001**	2.805 (1.998–3.937)	**<0.001**
(N3/N0)	3.526 (1.984–6.268)	**<0.001**	3.900 (2.239–6.792)	**<0.001**
TNM stage		**<0.001**		**<0.001**
(II/I)	2.796 (1.877–4.167)	**<0.001**	2.929 (2.005–4.279)	**<0.001**
(III/I)	7.397 (4.679–11.692)	**<0.001**	7.874 (5.083–12.198)	**<0.001**
Chemotherapy (no/yes)	1.578 (1.231–2.023)	**<0.001**	1.771 (1.396–2.247)	**<0.001**
Postoperative complications (yes/no)	3.617 (2.825–4.631)	**<0.001**	3.395 (2.666–4.324)	**<0.001**
Weekday (late/early)	1.574 (1.255–1.974)	**<0.001**	1.488 (1.201–1.844)	**<0.001**
Increased postoperative CRP (yes/no)	1.857 (1.490–2.315)	**<0.001**	1.856 (1.503–2.293)	**<0.001**

**Table 4:** Multivariate Cox regression analysis of overall and disease-free survival in 463 patients after gastrectomy.

Characteristics	Overall survival		Disease-free survival	
	HR (95% CI)	*p* value	HR (95% CI)	*p* value
Comorbidities (with/without)	—	—	0.886 (0.636–1.233)	0.472
Tumor size (≥5 cm/<5 cm)	—	—	1.055 (0.766–1.453)	0.741
Operation time (≥240 min/<240 min)	0.869 (0.582–1.299)	0.494	0.770 (0.520–1.139)	0.191
Operative blood loss (≥500 ml/<500 ml)	1.023 (0.691–1.514)	0.909	0.968 (0.662–1.417)	0.869
T classification		**0.017**		**0.018**
(T2/T1)	2.315 (0.902–5.945)	0.081	1.517 (0.670–3.433)	0.318
(T3/T1)	3.691 (1.373–9.921)	**0.010**	2.528 (1.062–6.020)	**0.036**
(T4/T1)	4.001 (1.231–13.007)	**0.021**	2.677 (0.920–7.789)	0.071
N classification		**<0.001**		**<0.001**
(N1/N0)	1.790 (1.350–2.374)	**<0.001**	1.988 (1.515–2.608)	**<0.001**
(N2/N0)	1.953 (1.144–3.333)	**0.014**	2.510 (1.515–4.157)	**<0.001**
(N3/N0)	2.838 (1.325–6.078)	**0.007**	2.954 (1.422–6.138)	**0.004**
TNM stage		**0.016**		**0.005**
(II/I)	1.182 (0.674–2.075)	0.560	1.118 (0.654–1.912)	0.684
(III/I)	2.520 (1.078–5.894)	**0.033**	2.536 (1.114–5.773)	**0.027**
Postoperative complications (with/without)	3.169 (2.388–4.204)	**<0.001**	2.826 (2.134–3.740)	**<0.001**
Weekday (late/early)	1.721 (1.343–2.205)	**<0.001**	1.693 (1.331–2.153)	**<0.001**
Chemotherapy (no/yes)	1.087 (0.830–1.423)	0.546	1.204 (0.928–1.562)	0.161
Increased postoperative CRP (yes/no)	1.399 (1.042–1.879)	**0.026**	1.533 (1.150–2.045)	**0.004**

**Table 5:** The death and relapse rate after gastric cancer were increased from Monday to Friday through Wednesday.

Weekday of surgery	*p* (OS)	HR (95% CI)	*p* (DFS)	HR (95% CI)
Monday	0.002	1	0.003	1
Tuesday	0.974	1.006 (0.695–1.457)	0.823	0.961 (0.677–1.364)
Wednesday	0.036	1.455 (1.025–2.065)	0.121	1.302 (0.933–1.819)
Thursday	0.001	1.724 (1.238–2.400)	0.001	1.722 (1.259–2.355)
Friday	0.005	1.559 (1.142–2.128)	0.021	1.415 (1.054–1.901)
