# Supplementary material for: Weekday of Surgery Affects Postoperative Complications and Long-Term Survival of Chinese Gastric Cancer Patients after Curative Gastrectomy
Source: Biomed Res Int. 2017 Apr 18;2017:5090534. doi: 10.1155/2017/5090534 (PMC5412209; doi:10.1155/2017/5090534)
Supplement: Supplementary file 1 — Degree of postoperative complication influenced the overall survival and disease-free survival. The occurance of postoperative complications promoted a much shorter survival time regardless of the weekday of surgery, however, the effect of weekday of surgery was limited in the group with complications. Even so, effect of weekday of surgery or postoperative complications was weak in the patients with advanced tumor stage for the little chance of cure. [file 5090534.f1.pdf]

Supplementary Table 1. When postoperative complication was categorized into the 5 degrees, the point HRs for Oversall survival(OS) and Disease-free survival(DFS) increased from Grade 0 to Grade IV.

Complications	*P*(OS)	HR(95%CI)	*P*(DFS)	HR(95%CI)
Grade 0	<0.001		<0.001	
Grade I	<0.001	3.169(2.275-4.415)	<0.001	3.006(2.169-4.166)
Grade II	<0.001	3.275(2.161-4.964)	<0.001	3.066(2.042-4.601)
Grade III	<0.001	5.547(3.340-9.213)	<0.001	5.196(3.133-8.619)
Grade IV	<0.001	5.328(2.889-9.828)	<0.001	4.875(2.648-8.975)

Supplementary Table 2. The effect of weekday of surgery was limited in the group with complications.

**Complications**	**Weekday of surgery**	***p*(OS)**	**HR(95%CI)**	***p*(DFS)**	**HR(95%CI)**
Without	Monday-Tuesday		1		1
	Wednesday-Friday	0.002	1.521(1.165-1.987)	0.006	1.415(1.102-1.817)
With	Monday-Tuesday		1		1
	Wednesday-Friday	0.208	1.319 (0.857-2.031)	0.188	1.333(0.869-2.045)

Supplementary Table 3. The occurance of postoperative complications promoted a much shorter survival time regardless of the weekday of surgery.

**Weekday of surgery**	**Complications**	***p*(OS)**	**HR(95%CI)**	***p*(DFS)**	**HR(95%CI)**
Monday-Tuesday	Without		1		1
	With	<0.001	3.889(2.562-5.903)	<0.001	3.468(2.315-5.194)
Wednesday-Friday	Without		1		1
	With	<0.001	3.304(2.427-4.499)	<0.001	3.139(2.316-4.254)

Supplementary Fig. 1

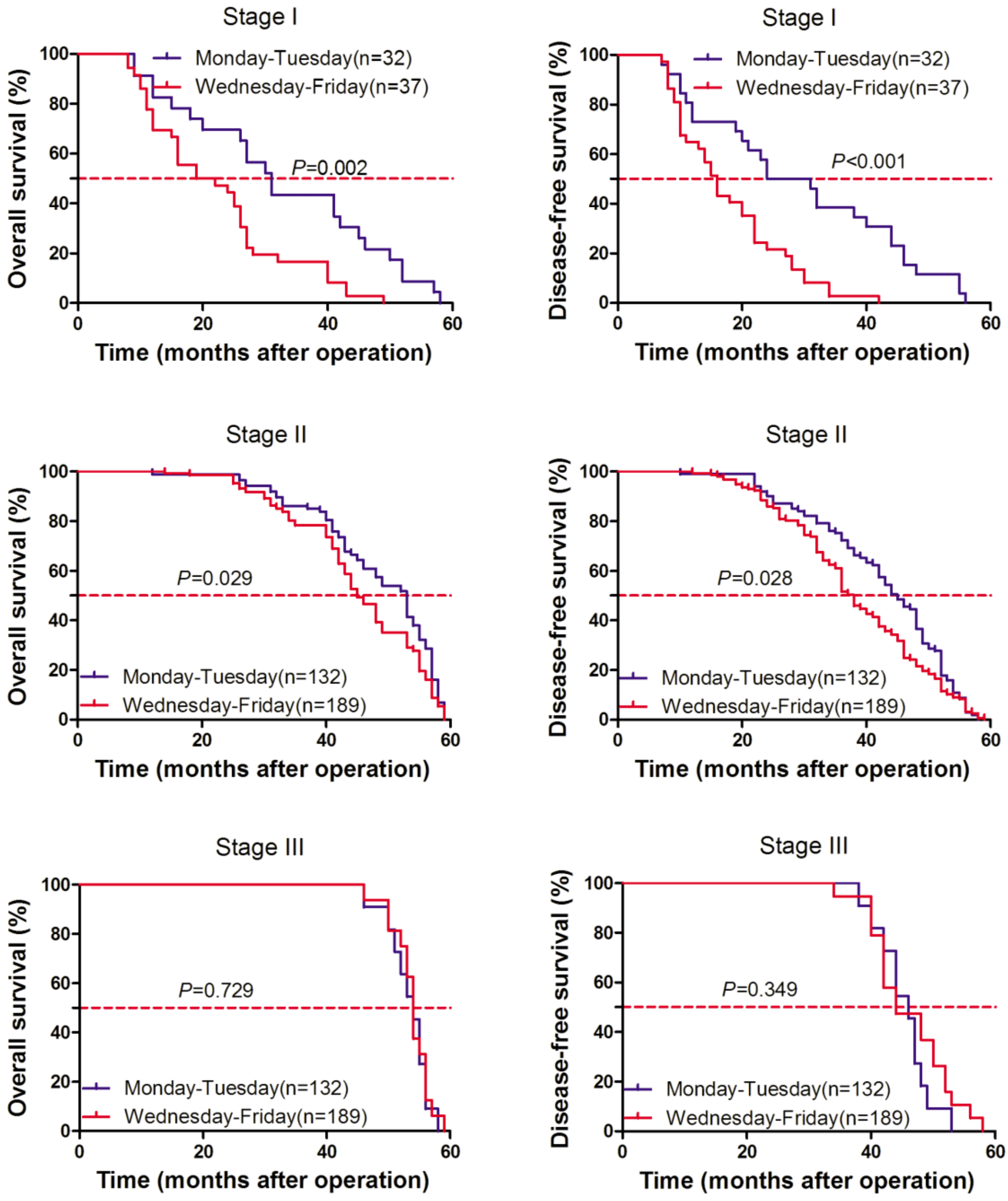

Legend for Supplementary Fig. 1

When stratified by the TNM stage, the influence of weekday of surgery was still evident in TNM stage I and II (A-D), but not in stage III (E, F).

Supplementary Fig. 2

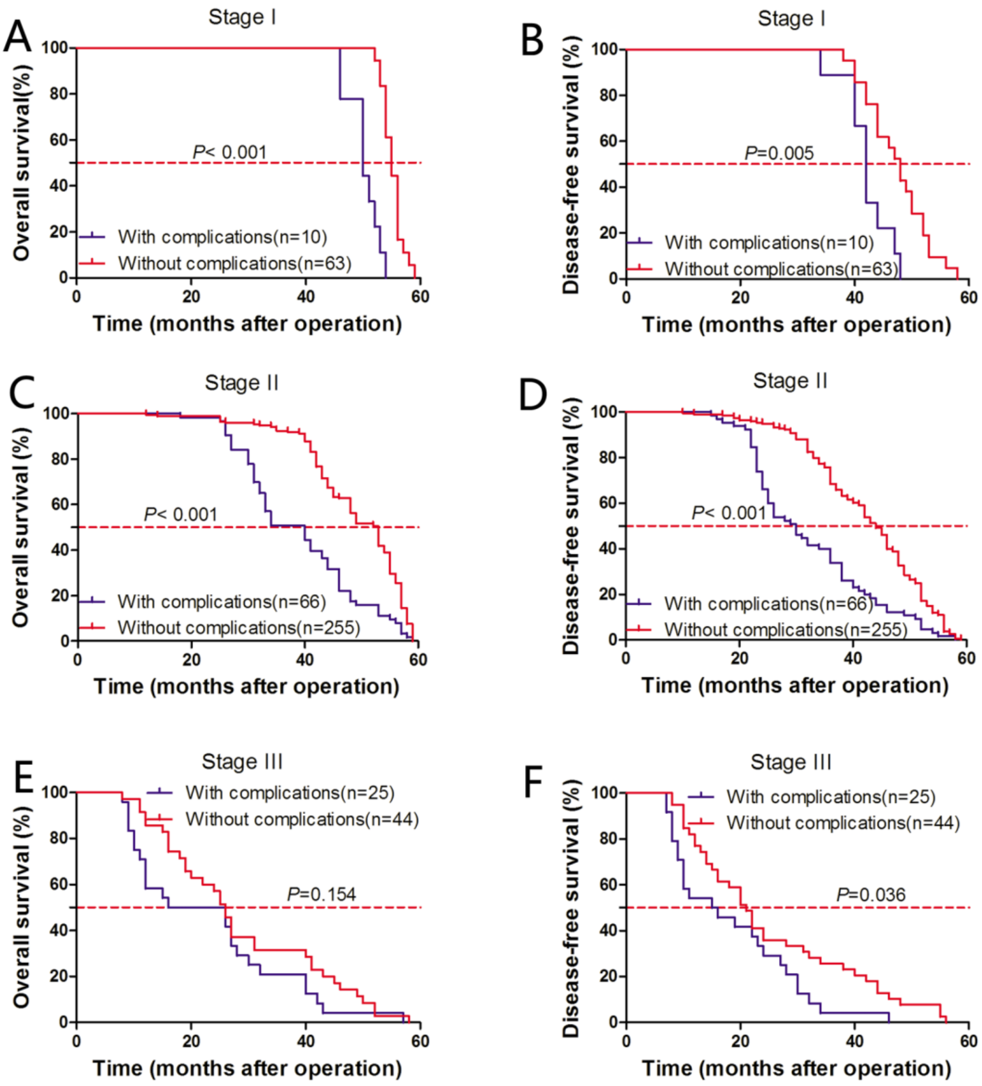

Legend for Supplementary Fig. 2

The influence of postoperative complications on overall survival and disease-free survival after gastric cancer surgery was displayed when stratified by the TNM stage. The postoperative complications promoted a shorter overall survival time, while increase the replase rate in TNM stage I and II(A-D). However, the 5-year overall survival rate was not shortened by the postoperative complications (E), while the disease-free survival time was effected significantly (F).
